# An Updated Review: Androgens and Cognitive Impairment in Older Men

**DOI:** 10.3389/fendo.2020.586909

**Published:** 2020-11-13

**Authors:** Zhonglin Cai, Hongjun Li

**Affiliations:** Department of Urology, Peking Union Medical College Hospital, Peking Union Medical College, Chinese Academy of Medical Sciences, Beijing, China

**Keywords:** androgen, cognitive impairment, aging, older men, molecular mechanism

## Abstract

Androgens are some of the most important sex hormones in men, and they maintain important physiological activities in the human body. Cognitive impairment is one of the most common manifestations of aging in the elderly population and an important factor affecting the quality of life of elderly individuals. The levels of sex hormones in elderly people decrease with age, and low levels of androgens in older male individuals have been closely linked to the development of cognitive impairment. Basic studies have shown that androgens have neuroprotective effects and that androgen deficiency impairs cognitive function by increasing oxidative stress and decreasing synaptic plasticity, among other effects. Additionally, clinical studies have also shown that androgen deficiency is closely related to cognitive impairment. This article reviews the relationship between low androgen levels and cognitive impairment, their potential mechanisms, and the effects of testosterone supplementation in improving cognition.

## Introduction

Human aging is an inevitable process (1). As aging occurs, the gonads decrease in function, and there is a decrease in the levels of sex hormones (2). The most important sex hormones in men are androgens, which are involved in maintaining the normal functions of multiple tissues and organs in human physiology (3). In older men, in addition to their effects on the reproductive system, low levels of androgens have been reported to increase the risk for many aging-related diseases in other systems, such as tumors, diabetes mellitus and cardiovascular diseases (4, 5). Therefore, androgens are vital to older men.

Androgens mainly include testosterone produced by Leydig cells, the testosterone metabolite dihydrotestosterone (DHT), adrenal-derived dehydroepiandrosterone (DHEA), androstenedione, androstenediol, and androstenone. The brain can synthesize neurosteroids using peripheral sex hormones, which can also be synthesized *de novo* by using cholesterol in the brain (6, 7). Neurosteroids synthesized in the brain include androgens such as testosterone, 3*α*-androstanediol, DHEA, and allopregnanolone, which play an important role in the maintenance of brain function (8). Androgens can improve the biological activity of cells by increasing the production of ATP in the mitochondria and can also increase antioxidant activity to regulate redox homeostasis, thereby exerting a neuroprotective effect (9, 10). The cerebral vasculature is also the target of androgens, which increase vascular tone, enhance angiogenesis and cerebral vascular remodeling, reduce vascular damage by attenuating oxidative stress, and maintain the integrity of the blood–brain barrier, thereby exerting additional neuroprotective effects (11). In the hippocampus of the brain, testosterone can improve synaptic plasticity and prevent neuronal cell death. DHT induces circuit modifications by changing the number of excitatory spine synapses in a paracrine manner, which in turn affects the cognitive function of the brain (12).

Cognitive function is the result of high-level neural activity in the human brain. It mainly involves verbal, spatial and memory ability (13). Therefore, the manifestations of cognitive impairment are diverse and often appear in multiple forms. Among women, although androgen is not main sex hormone, it has been demonstrated that androgen deficiency is closely related to cognitive impairment (14). For elderly men, in addition to brain aging causing cognitive dysfunction (15), low androgen levels caused by aging can further aggravate cognitive decline in the brain (16). Therefore, in older men, low levels of androgens and cognitive impairment also have a close relationship. This article will objectively discuss the relationship between androgens and cognitive function in older men, potential mechanisms, and the roles of testosterone supplementation in improving cognition.

## Methods

The narrative review was conducted on extracted and summarized findings from relevant literature by an electronic search of the PubMed databases. The electronic search was performed according to the following keywords: (androgen OR testosterone OR hypogonadism) AND (cognition OR dementia OR Alzheimer’s disease). Based on the aim of this narrative review, the findings published after 2000 were recurrently reviewed by considering title and abstract and evaluating full texts, and relevant studies were selected. Additional studies were also selected from the reference lists in selected studies.

### Relationship Between Decreased Androgens and Impaired Cognition in the Normal Aging Process

Sex hormones are regulated and produced by the gonads through the gonadal (hypothalamus–pituitary–testis) axis in males. Any factors that affect the gonadal axis, including aging and gonadal-related diseases, can cause an imbalance in the levels of sex hormones, such as testosterone, DHEA, luteinizing hormone (LH) and follicle-stimulating hormone (FSH). Hypogonadism is a clinical syndrome due to androgen deficiency caused by failure of testicular function, and its incidence was 38.7% in males aged >45 years (total testosterone level <300 ng/ml) (17). Clinically, hypogonadism due to aging is called late onset hypogonadism. It has been reported that the incidence of testosterone deficiency is approximately 20% in men aged 60 years and increases to 50% in 80-year-old men (18). Therefore, androgen deficiency caused by aging cannot be ignored due to the high incidence in older men. Although aging can directly lead to cognitive decline, androgen deficiency is considered to be another important cause of cognitive decline in older men (19). In studies of cognitive function evaluated by the MMSE and sex hormone levels in older men, the levels of sex hormones, including testosterone, DHT, estradiol (E2), estrone (E1), LH, and FSH, change over time (20). These changes have been related to declines in cognitive ability (20). However, interestingly, only serum testosterone, DHT and calculated FT are associated with decreased cognitive function after correcting for confounding factors (20). This finding demonstrated that androgen levels are closely related to cognitive impairment in elderly men.

Various types of androgen have been found to be associated with cognition function. A meta-analysis of seven prospective cohort studies has shown that low levels of plasma testosterone are significantly associated with an increased risk of AD in older men (RR = 1.48, 95% CI 1.12–1.96, P = 0.006) (21). In addition to total testosterone (TT), plasma DHT, and FT in elderly men are also significantly correlated with cognitive decline according to the MMSE score (20). It has been shown that the decrease in TT and FT levels in plasma, which is independent of the relevant clinical and biochemical factors, is associated with an increased risk for dementia in older men (22), and the risk ratios of TT and FT for dementia were 1.14 and 1.18, respectively (23). Additionally, despite a lack of research on endogenous DHT, which can be converted from testosterone, and cognitive dysfunction, 5*α* reductase inhibitor-induced DHT deficiency has been shown to increase the risk of dementia in elderly men (24), suggesting that testosterone deficiency may lead to a lack of DHT, which is closely related to cognitive dysfunction. DHEA and its sulfated form (DHEA-S) are important neurosteroids that regulate brain development and function. A correlation between serum DHEA-S levels and working memory has been found to be strong in males (25). Thus, androgen plays an important role in cognitive function, and low androgen levels are an important cause of cognitive impairment in older men.

SHBG is the main transport for testosterone and regulation of total testosterone bioactivity (26). In older men, with increasing age, the level of SHBG increases. Additionally, compared with healthy aging males, the level of SHBG in elderly AD patients increased significantly (27). However, levels of SHBG have no relationship with cognitive decline in patients with cognitive impairment according to the Alzheimer’s Disease Assessment Scale-Cognitive section (28). Interestingly, it has been found that in aging males, decreased bioavailable testosterone (BT), a non-SHBG-bound form of testosterone, is closely correlated with cognitive function according to the Standardized Mini Mental Test for the diagnosis of dementia (26). Low BT was associated with a greater risk for dementia (HR 1.29, 95% CI 1.03–1.62, P < 0.01), and the above relationship is stronger in men over 80 years of age (29). Therefore, with aging, SHBG levels increase, BT may be a neglected important cause of cognitive impairment in older men.

### More Clinical Proof for Androgen Reduction and Cognitive Impairment

Accumulative studies have found that androgen levels decrease in the context of a variety of diseases and corresponding treatments, including schizophrenia, acquired immune deficiency syndrome, multiple sclerosis, hypogonadism, and acute lymphoblastic leukemia (23, 30–32). These clinical studies have also found that these populations with low androgen levels have a higher risk of cognitive impairment, such as spatial memory and attention deficit. As a common disease in elderly patients, type 2 diabetes mellitus has been shown by an increasing number of studies to lead to lower testosterone levels in men, increase testosterone resistance, and increase the incidence of Alzheimer’s disease (AD), a serious cognitive impairment disease (33).

Prostatic hyperplasia is a common disease in older men and often causes lower urinary tract symptoms (LUTS). 5*α* reductase inhibitors are commonly used in the treatment of male prostatic hyperplasia. They can reduce the level of DHT. The risk for dementia in patients on 5*α* reductase inhibitors is 2.18 and 1.52 times that of patients in the first and second years, respectively (24). This suggests that 5*α* reductase inhibitors may increase the risk of dementia by reducing the level of DHT.

Prostate cancer (PCa) is a common cancer in elderly men. ADT is an important treatment for inhibiting the growth of PCa that functions by lowering testosterone levels. Significant neurophysiological and psychological problems occur after ADT (34). Accumulative studies have shown that compared with those of patients without ADT, the cognitive functions, including language ability, short-term memory, prospective memory, mental flexibility, inhibitory control and emotional psychology, of patients with ADT are significantly impaired (34–39). After ADT treatment, cognitive impairment occurs within 6 and 12 months, and the risk for dementia is greatest when ADT exceeds 12 months (38, 39). Compared with non-ADT patients, the risk for dementia in patients with ADT increases by 4.4 and 5.8% within follow-up periods of 5 and 8.3 years, respectively (38–40). Additionally, generally, the higher the dose of ADT is, the higher the risk of dementia (40). AD is a serious type of dementia-related disease. Multiple studies have also shown that AD risk in the ADT group is 1.14–1.84 times higher than that in the non-ADT group after adjusting for confounding factors (40–42). Patients over 70 years of age have the greatest risk for AD after ADT compared with patients under 70 years of age (43). However, due to heterogeneity of general characteristics and study methods, some studies have shown that ADT is not associated with dementia or AD (44–48). A recent meta-analysis with high evidence grade confirmed that ADT increased the risk for dementia in PCa patients (49), suggesting that low androgen derived from ADT is an important cause of cognitive dysfunction. Additionally, changes in tissue structure after ADT support cognitive dysfunction. It has been found that ADT treatment can induce a significant reduction in gray matter volume and an increase in white matter lesion load in multiple regions, including the frontopolar cortex, dorsolateral prefrontal cortex, and primary motor cortex (50, 51). Thus, cognitive set shifting is impaired, and responses evoked by ventral hippocampus afferents are attenuated, resulting in cognitive dysfunction (52). Therefore, low androgen caused by ADT is closely related to cognitive impairment in male patients.

### Mechanism of Low Androgen-Induced Cognitive Impairment

In the AD rat model produced by intraventricular injection of streptozotocin, there is severe deterioration in the memory of the rats after testicular castration, and this memory could be significantly restored by supplementation with testosterone (53), suggesting that testosterone can improve cognitive and memory disorders. Androgen plays biological roles in the brain mainly by binding to androgen receptors (ARs), which are widely expressed in multiple brain regions, including the hippocampus and prefrontal cortex (54). Clinical AD samples have shown that AD patients have multiple single nucleotide polymorphisms in ARs in multiple tissues, including the brain and that the level of AR expression is reduced (30). It has further been suggested that androgen binds to ARs in the brain tissue and thus affects cognitive function.

**Figure 1:**
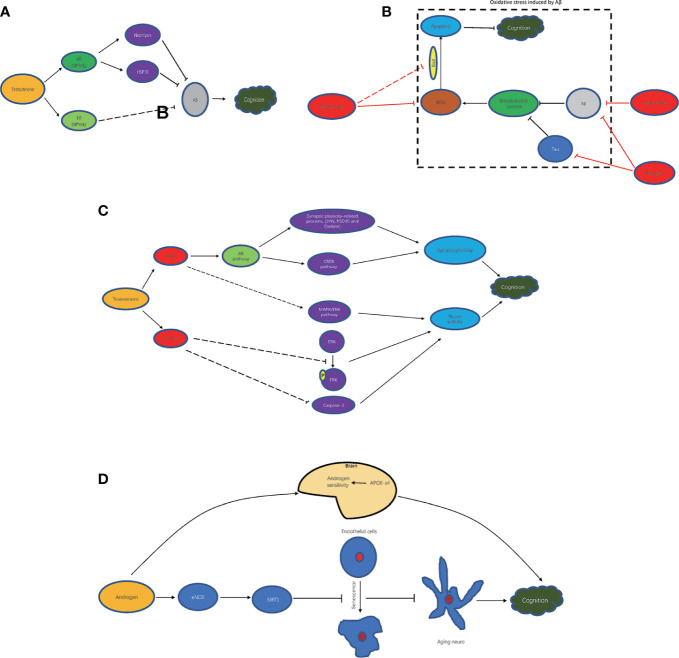
Mechanism of androgen improving cognitive impairment. **(A)** androgen and A*β* clearance; **(B)** androgen and oxidative stress inhibition; **(C)** androgen metabolites and cognition improvement; **(D)** androgen-related other mechanism.

Androgen is closely related to brain beta amyloid (A*β*) by the AR signaling pathway (55). Reduced androgen levels induce A*β* accumulation in brain neurons. A*β* is an important pathological molecule in the brain tissue of patients with AD and impairs neurons. Hippocampal neurons and their synaptic plasticity are the basis of spatial memory and cognition. Accumulative A*β* in hippocampal neurons directly disrupts cognition by complicated pathological mechanisms that induce abnormal synaptic structures, synaptic plasticity reduction, and neuroviability impairment (10, 56). It has been found that in male rats treated with testosterone, the expression of A*β*1–42 protein was significantly decreased. The mechanisms by which testosterone regulates A*β* levels in neurons are as follows: 1. Elevating neprilysin to accelerate A*β* clearance (57); 2. Inhibiting A*β* accumulation by increasing HSP70 (56, 58). In addition to the AR signaling pathway, testosterone can clear A*β* by the estrogen receptor signaling pathway (59). In summary, based on the above mechanisms, androgen contributes to improving cognitive function by improving A*β*-mediated impairment of hippocampal neurons by reducing A*β* accumulation **(** **Figure 1A** **)**.

It is known that increases in oxidative stress can seriously damage brain function (60, 61). The levels of reactive oxygen species (ROS) and the number of apoptotic cells in brain tissue are significantly increased in diet-induced obese rats, while the levels of ROS and the number of apoptotic cells are further increased after orchidectomy (ORX) treatment (60). After ORX treatment, the levels of oxidative stress molecules, such as ROS and malondialdehyde, are significantly increased in the hippocampus, leading to the overactivity of glial cells and apoptosis and then hippocampal dysplasia and cognitive decline (62). In rats with intact gonads, chronic intermittent hypoxia (CIH) induces increased levels of oxidative stress in AD-related brain regions, which leads to memory impairments (63). It has been shown that testosterone scavenges free radicals to enhance synaptic plasticity by the AR signaling pathway in the hippocampus (64). In addition, A*β* can impair mitochondrial function and induce increased oxidative stress. Testosterone could attenuate mitochondrial dysfunction-induced A*β* and estrogen, which is converted from testosterone, and could improve mitochondrial dysfunction in hippocampal neurons of AD animals, which is induced by A*β* or tau protein (65), another important pathological molecule of AD. Conversely, testosterone supplementation can preserve memory function and prevent CIH-induced oxidative stress changes (63). Testosterone could inactivate Bcl-2-related dead cells (Bad) and inhibit apoptosis of cerebellar granule cells induced by oxidative stress, thus alleviating cognitive impairment (66–68). Thus, testosterone can improve cognitive impairment by reducing mitochondrial damage and oxidative stress in neurons **(** **Figure 1B** **)**.

Testosterone metabolites such as DHT, 5*α*-androstane-3*α*,17*β*-diol (3*α*-diol) also participate in neuroprotection **(** **Figure 1C** **)**. DHT enhanced synaptic plasticity-related proteins, such as SYN, PSD95, and Drebrin, and activated the CREB signaling pathway by AR, thereby improving the hippocampal synaptic structure and increasing synaptic plasticity to improve cognitive function (69). Additionally, it also contributed to improving neuroviability by activating the MAPK/ERK signaling pathway. 3*α*-Diol inhibited the delay in ERK phosphorylation and caspase-3 caused by A*β* 42 and reduced all the cellular activity induced by A*β* or hydrogen peroxide, thus exerting neuroprotective effects (70).

In addition to the above mechanism, androgen can also improve cognitive function through other molecules and corresponding pathways **(** **Figure 1D** **)**. In carriers of APOE-*ϵ*4, APOE-*ϵ*4 increases the sensitivity of the brain to testosterone, thereby increasing tolerance to the effects of aging produced by lower testosterone levels that lead to cognitive decline (71). In addition, it has shown that aging endothelial cells in the brain can promote the aging of neurons. In aging vascular endothelial cells, testosterone induces the expression of SIRT1 by enhancing eNOS activity to inhibit the aging of endothelial cells, thereby inhibiting the aging of neurons and slowing cognitive decline (72).

Thus, basic studies have shown that testosterone, as the main androgen, plays an important neuroprotective role in the brain, especially in the hippocampus. The decrease in androgen levels may be one of the important reasons for cognitive impairment in elderly patients.

### Is There a Need for Testosterone Supplementation?

Basic studies, as reviewed above, have confirmed that testosterone levels are associated with cognitive impairment and that testosterone supplementation has obvious neuroprotective effects. Most clinical studies have also supported the association between low testosterone levels and cognitive impairment. However, clinical studies on testosterone supplementation and improvements in cognitive function in elderly men are inconsistent.

For older men with low testosterone and cognitive impairment, two small-sample studies have shown that testosterone supplementation (50–100 mg of testosterone daily) results in improvements in global cognition, verbal memory and depressive symptoms (73, 74). In another study with 106 elderly patients with testosterone deficiency syndrome, after 8 months of testosterone replacement treatment (injection with 1,000 mg testosterone undecanoate), the total serum testosterone levels significantly increased, depression scores significantly decreased, and cognitive function scores according to the MMSE significantly increased (75). These studies all showed that testosterone supplementation contributes to improving cognitive impairment. However, a large-sample prospective study showed that after supplementation with testosterone (1% testosterone gel, 5 g daily) for 12 months in older men with low testosterone levels and age-related cognitive impairment, there was no significant increase in delayed paragraph recall, visual memory, executive functioning and spatial ability, suggesting that testosterone supplementation was not related to improvements in cognitive functioning (76). The heterogeneity of the above results may be due to different cognitive evaluations, sample sizes, study designs, and methodologies, *etc*. Meanwhile, a recent meta-analysis of testosterone supplementation in middle-aged or aging males with cognitive impairment or dementia also failed to confirm that testosterone supplementation contributes to cognitive improvement (77). Additionally, for older men with low androgen and without cognitive impairment, most studies did not provide supporting evidence that testosterone supplementation can enhance cognition (78–80). Interestingly, a meta-analysis of testosterone supplementation and the prevention of cognitive decline showed significant improvements in overall cognitive composite scores, psychomotor speed and executive function in elderly patients with testosterone supplementation (81). In short, although testosterone deficiency is closely related to cognitive impairment, testosterone supplementation for improving cognitive function in aging males remains further clarified.

## Conclusions

With aging, declines in cognitive function are one of the important factors affecting the quality of life of elderly individuals. Most clinical studies have confirmed that there is a close relationship between low androgen levels and cognitive impairment in elderly individuals; meanwhile, basic research has shown that testosterone contributes to the maintenance of cognitive function, which is further supported by corresponding mechanistic research. Androgen supplementation seems to be a promising treatment for improving cognitive impairment in older men. However, most of the existing clinical studies about androgen supplementation do not support that androgen supplementation can be helpful for cognitive improvement in older men (69–72). Together, androgen deficiency is an important cause of cognitive impairment in older men and should receive attention. More studies of testosterone supplementation in improving cognition are needed.

## Author Contributions

ZC collected all related references and drafted the manuscript. HL was responsible for the conception and design of the work. All authors contributed to the article and approved the submitted version.

## Funding

This study was funded by the grant from National Natural Science Foundation of China (Grant No. 81871152).

## Conflict of Interest

The authors declare that the research was conducted in the absence of any commercial or financial relationships that could be construed as a potential conflict of interest.
